# Machine Learning‐Based Immuno‐Inflammatory Index Integrating Clinical Characteristics for Predicting Coronary Artery Plaque Rupture

**DOI:** 10.1002/iid3.70162

**Published:** 2025-04-07

**Authors:** Xi Wang, Qianhang Xia, Shuangya Yang, Chancui Deng, Ning Gu, Youcheng Shen, Zhenglong Wang, Bei Shi, Ranzun Zhao

**Affiliations:** ^1^ Department of Cardiology Affiliated Hospital of Zunyi Medical University Zunyi China; ^2^ Department of Cardiology The Third Affiliated Hospital of Zunyi Medical University (The First Peoples Hospital of Zunyi) Zunyi China

**Keywords:** acute coronary syndrome, machine learning, optical coherence tomography, systemic inflammation index, systemic inflammation response index

## Abstract

**Background:**

Coronary artery plaque rupture (PR) is closely associated with immune‐inflammatory responses. The systemic inflammatory index (SII) and the systemic inflammatory response index (SIRI) have shown potential in predicting the occurrence of PR.

**Objective:**

This study aims to establish a machine learning (ML) model that integrates baseline patient characteristics, SII, and SIRI to predict PR. The goal is to identify high‐risk PR patients before intravascular imaging examinations.

**Methods:**

We included 337 patients with acute coronary syndrome who underwent emergency percutaneous coronary intervention and coronary optical coherence tomography (OCT) at the Affiliated Hospital of Zunyi Medical University, China, from May 2023 to October 2023. PR was determined by OCT images. Through manual feature selection, nine features, including SII and SIRI, were included, and an ML model was built using the XGBoost algorithm. Model performance was evaluated using receiver operating characteristic curves and calibration curves. SHAP values were used to assess the contribution of each feature to the model.

**Results:**

The ML model demonstrated a higher area under the curve value (AUC = 0.81) compared to using SII or SIRI alone for prediction. The ML model also showed good calibration. SHAP values revealed that the top three features in the ML model were SII, LDL‐C, and SIRI.

**Conclusion:**

The immuno‐inflammatory index, which integrates comprehensive clinical characteristics, can predict the occurrence of PR. However, large‐scale, multicenter studies are needed to confirm the generalizability of the predictive model.

## Graphical abstract

We developed and validated a novel machine learning model that integrates multidimensional features, including SII and SIRI, to predict the occurrence of PR. Our study highlights the potential of ML in predicting PR occurrence, offering promise in assisting physicians to identify high‐risk patients promptly.

## 1 Background

The global incidence of cardiovascular diseases (CVD) continues to rise, making it the leading cause of death worldwide [1]. Coronary artery plaque rupture (PR) or erosion is a critical mechanism underlying acute coronary syndrome (ACS) [2]. Coronary plaques are formed by the accumulation of lipids, cholesterol, calcium, and other substances on the arterial wall [3]. When PR occurs, the internal lipid core is exposed to the bloodstream, triggering platelet aggregation and coagulation, leading to thrombus formation [4]. This can partially or completely occlude the coronary artery, resulting in myocardial ischemia or infarction, and subsequently causing ACS.

Atherosclerosis development is driven by four major factors: the immune system, inflammatory processes, lipid metabolism abnormalities, and CVD risk factors [5, 6]. Inflammation and immune responses play a crucial role in the occurrence and progression of PR [7, 8]. Inflammatory cells, such as macrophages and T cells, infiltrate the plaque, releasing various inflammatory mediators and enzymes that weaken the fibrous cap of the plaque, making it vulnerable and ultimately leading to PR [9, 10].

The systemic inflammatory index (SII) and the systemic inflammatory response index (SIRI) are novel indicators reflecting systemic inflammatory responses and immune status [11]. Recent findings have linked SII and SIRI with cardiovascular diseases and all‐cause mortality risk [12, 13]. Evidence suggests that elevated levels of SII and SIRI may adversely affect plaque vulnerability [14, 15], making them potential predictive features for PR.

However, the occurrence of PR is a complex result of multiple pathophysiological processes. Predicting PR based on a single indicator may not capture its multidimensional characteristics. In recent years, ML algorithms have shown significant potential in integrating multidimensional features and predictive modeling [16].

In this study, we aim to explore the application of ML algorithms in predicting PR. By integrating data from multiple dimensions, including patient baseline characteristics, SII, and SIRI, we strive to develop a predictive model capable of identifying patients at high risk of PR. This will assist clinicians in better assessing patient risk, enabling early and effective intervention measures for high‐risk patients, thereby improving patient survival rates and quality of life.

## 2 Methods

### 2.1 Patient Selection

This retrospective observational study included 337 patients with ACS who underwent coronary angiography and optical coherence tomography (OCT) at the Affiliated Hospital of Zunyi Medical University, China, from May 2023 to October 2023. Among them, there were 279 males (82.8%) and 58 females (17.2%), with a mean age of (61.50 ± 10.96) years. Inclusion criteria were as follows: (1) patients with symptoms of chest pain; (2) underwent coronary angiography and OCT examination; (3) complete clinical data available. Exclusion criteria included: (1) patients with restenosis due to stent; (2) concomitant myocarditis, pericarditis, cardiomyopathy, congenital heart disease, valvular heart disease, or other organic heart diseases; (3) poor image quality hindering analysis. This study was approved by the Ethics Committee of Zunyi Medical University.

### 2.2 Data Collection and Laboratory Tests

Baseline clinical characteristics, including age, gender, medical history, and smoking status, were collected by physicians using the electronic medical record system under confidentiality. Peripheral venous blood samples (2 mL) were collected on the morning of the first day of hospitalization for analysis.

### 2.3 Definition of SII and SIRI

SII was calculated as (neutrophil count) × (platelet count)/(lymphocyte count). SIRI was calculated as (neutrophil count) × (monocyte count)/(lymphocyte count).

### 2.4 PCI and Postoperative Medication

All PCI procedures were performed by the same 2–3 experienced interventional cardiologists. Before emergency surgery, patients orally received enteric‐coated aspirin 300 mg and clopidogrel 300 mg. For elective surgery, patients received aspirin 100 mg/d and clopidogrel 75 mg/d for three consecutive days. During the procedure, patients were in a supine position, and radial or femoral artery puncture was performed using the Seldinger technique. Subcutaneous infiltration anesthesia with lidocaine was used at the puncture site. During the operation, 8000–10,000 U of heparin (depending on body weight) was administered through the arterial sheath for anticoagulation. Postoperatively, patients received subcutaneous injection of 4000 IU of low molecular weight heparin twice daily for 3–5 days, along with oral aspirin 100 mg once daily and clopidogrel 75 mg once daily, continued for at least 12 months. Treatment success was defined as residual stenosis < 10% observed in at least two orthogonal projection positions, distal vessel with TIMI 3 flow, and absence of procedure‐related serious complications (e.g., myocardial infarction, sudden death, and emergency CABG).

### 2.5 OCT Image Acquisition and Feature Analysis

Vascular OCT imaging was conducted using commercially available systems (ILUMIEN OPTIS, OPTIS Integrated, and OPTIS Mobile systems; Abbott Vascular), which include rapid‐exchange catheters (Dragonfly DUO, Dragonfly OPTIS, Dragonfly OpStar imaging catheters; Abbott Vascular) and integrated pullback systems (18–36 mm/s), acquiring images with high (~15 μm) axial resolution to depict blood displacement. If necessary, images were obtained post‐dilation and after intracoronary nitroglycerin administration. All OCT images were analyzed independently by two observers to identify plaque rupture. The criteria for PR diagnosis were based on the presence of a fibrous cap tear with underlying thrombus, as observed in the OCT imaging. Specifically, a disruption of the fibrous cap, often with a cavity and intraplaque hemorrhage or thrombus, was required for the classification of PR. Non‐plaque rupture (NPR) was defined as the absence of a fibrous cap tear, with stable or minimally disrupted plaque morphology, without significant intraplaque hemorrhage or thrombus. For patients with severe calcified lesions who did not undergo rotational atherectomy, cases where fibrous cap rupture was not observed were classified as NPR.

### 2.6 Prediction Model Construction

Logistic regression models were separately constructed using SII and SIRI to predict PR. Employ LASSO regression for feature selection based on patients' baseline characteristics and laboratory test data. Utilize the key features identified through LASSO regression to construct a machine learning prediction model. Extreme Gradient Boosting (XGBoost) was utilized for modeling. Patients were randomly assigned to training and validation sets at a ratio of 3:1, employing fivefold cross‐validation and grid search to optimize parameters. Detailed algorithm parameters used in our analysis are provided in Supporting Material Table 1.

### 2.7 Statistical Analysis

Kolmogorov‐Smirnov test was used to assess variable distribution. Continuous variables were presented as mean ± standard deviation or median with interquartile range (IQR), and compared using *t*‐test or Mann‐Whitney U test based on data distribution. Categorical variables were expressed as counts and percentages, and compared using Fisher's exact test or Chi‐square test as appropriate. Receiver operating characteristic (ROC) curves were plotted, and area under the curve (AUC) was calculated to evaluate the model's predictive value for PR. Accuracy, sensitivity, specificity, positive predictive value (PPV), negative predictive value (NPV), and F1 score were computed as follows: Sensitivity = True positive cases/(True positive cases + False negative cases), Specificity = True negative cases/(True negative cases + False positive cases), PPV = True positive cases/(True positive cases + False positive cases), NPV = True negative cases/(True negative cases + False negative cases), Accuracy = (True positive cases + True negative cases)/(True positive cases + False positive cases + True negative cases + False negative cases). Statistical analysis was conducted using Python programming language version 3.9. A *p*‐value < 0.05 was considered statistically significant.

## 3 Results

**Figure 1:**
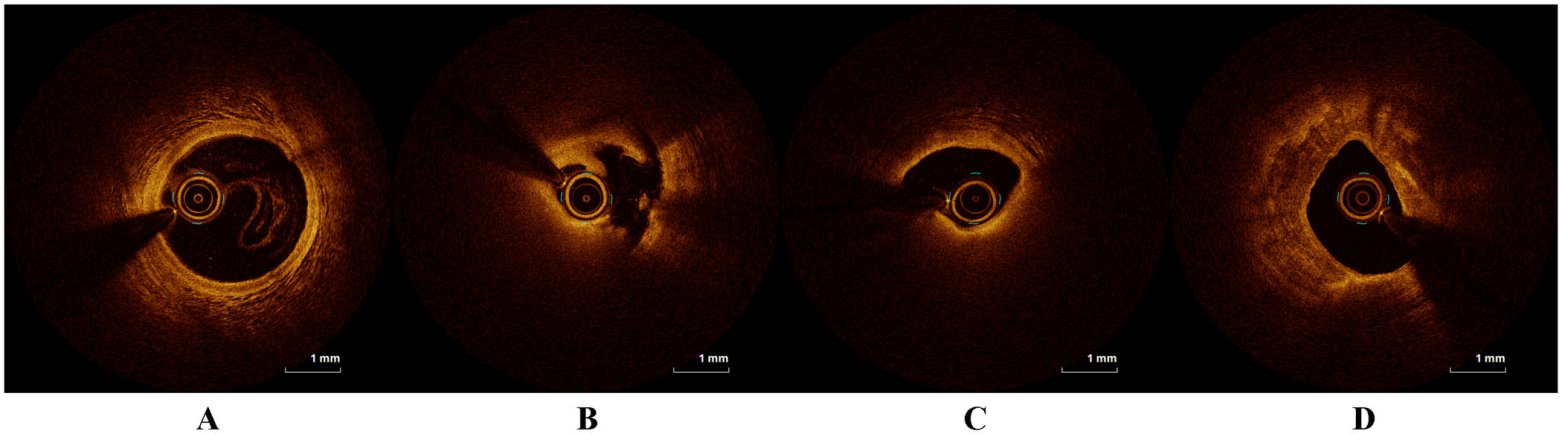
Partial OCT Images. (A) Normal coronary OCT image; (B) plaque rupture; (C) Lipid plaque; (D) Calcified plaque.

**Figure 2:**
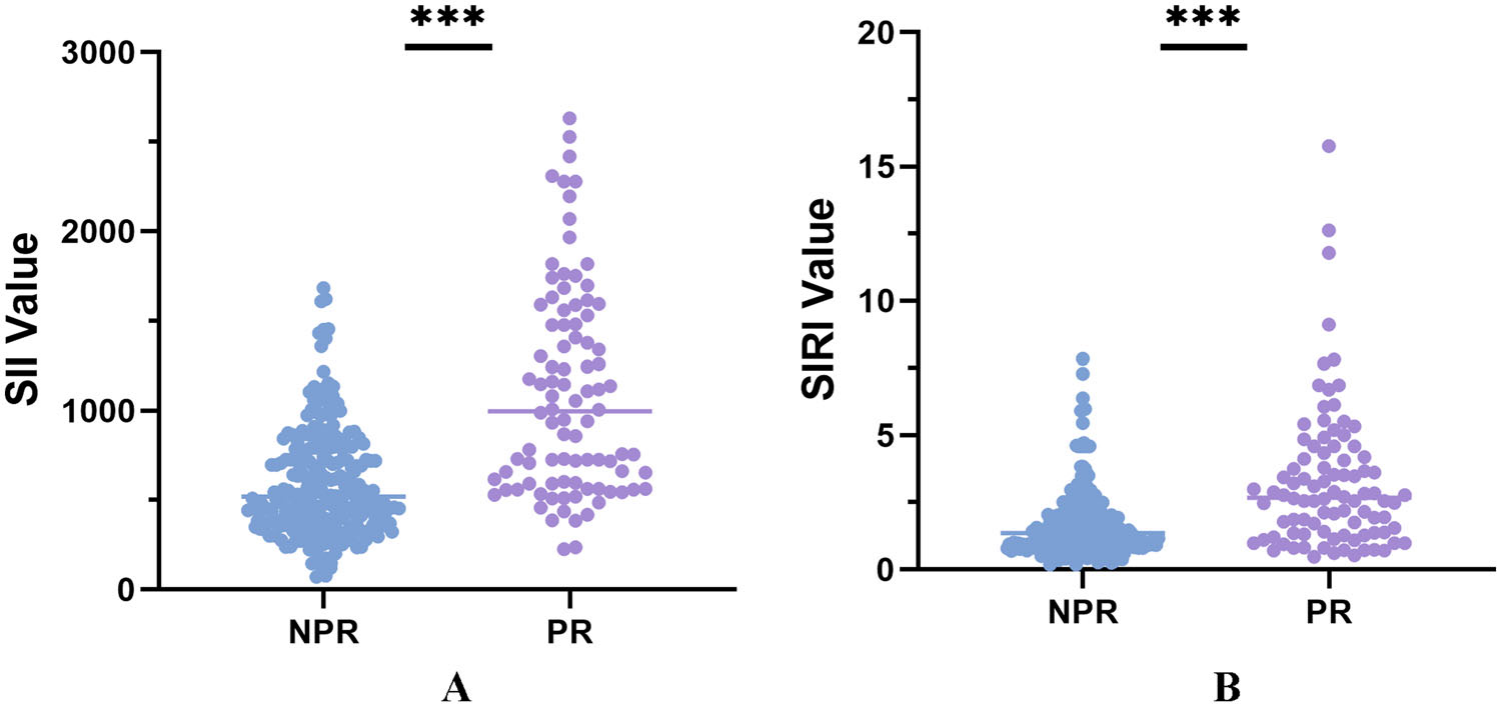
The difference in SII and SIRI between the two groups of patients. (A) The SII Value; (B) The SIRI Value.

A total of 337 patients were included in the study, and the detailed patient selection flowchart is provided in Supporting Material Figure 1. Examples of OCT images of partial lesions are shown in Figure 1. Clinical characteristics between NPR and PR groups are summarized in Table 1. There were no statistically significant differences between the two groups in baseline characteristics such as age, gender, and smoking history. However, in laboratory examinations, the PR group exhibited higher levels of LDL‐C (*p* = 0.008, Table 1) and TC (*p* = 0.045, Table 1) compared to the NPR group. Regarding inflammatory markers, the PR group showed higher white blood cell count (*p* < 0.001, Table 1), neutrophil count (*p* < 0.001, Table 1), and monocyte count (*p* = 0.009, Table 1) than the NPR group. Although there was no statistical difference in lymphocyte count between the two groups (*p* = 0.068, Table 1), the PR group demonstrated higher SII and SIRI (*p* < 0.001), as depicted in Figure 2. There was a significant difference in the distribution of SII and SIRI between the two groups. Additionally, cardiac troponin levels showed a statistically significant difference (*p* < 0.001) between the two groups. In cardiac ultrasound examination, the PR group had relatively lower EF values (*p* = 0.022).

**Table 1:** Characteristics of Participants.

		NPR (*n* = 245)	PR (*n* = 92)	*p*
**Demographics**				
Age (years)		62.00 [54.00, 70.00]	59.50 [51.00, 69.25]	0.150
Gender (%)	Female	42 (17.1)	16 (17.4)	1.000
	Male	203 (82.9)	76 (82.6)	
BMI (kg/m^2^)		25.32 [22.98, 26.95]	25.39 [23.34, 27.34]	0.293
**Clinical features**				
HR (BPM)		76.00 [70.00, 86.00]	76.00 [70.00, 89.25]	0.700
SBP (mmHg)		129.00 [115.00, 142.00]	128.00 [107.00, 140.00]	0.319
DBP (mmHg)		79.00 [70.00, 89.00]	78.00 [71.75, 89.25]	0.926
Hypertension (%)	N	107 (43.7)	38 (41.3)	0.789
	Y	138 (56.3)	54 (58.7)	
Diabetes (%)	N	193 (78.8)	75 (81.5)	0.685
	Y	41 (21.4)	16 (27.1)	
Smoker (%)	N	106 (43.3)	37 (40.2)	0.703
	Y	139 (56.7)	55 (59.8)	
**Laboratory data**				
TG (mmol/L)		1.73 [1.16, 2.63]	1.73 [1.12, 2.79]	0.663
TC (mmol/L)		4.80 [3.82, 5.48]	5.03 [4.37, 5.65]	0.045
HDL‐C (mmol/L)		1.11 [0.95, 1.27]	1.10 [0.96, 1.24]	0.967
LDL‐C (mmol/L)		2.83 [2.08, 3.40]	3.09 [2.62, 3.60]	**0.008**
Fasting glucose (mmol/L)		6.20 [4.94, 8.00]	6.72 [5.21, 9.31]	0.058
hs‐TNT (ng/L)		14.05 [7.59, 60.14]	99.69 [11.50, 484.70]	**< 0.001**
WBC count (10^9^ cells/L)		6.62 [5.47, 8.07]	8.91 [6.90, 11.96]	0.214
RBC count (10^9^ cells/L)		4.57 [4.20, 4.97]	4.58 [4.15, 5.05]	0.970
Platelet count (10^9^ cells/L)		197.00 [156.00, 231.00]	217.50 [172.50, 272.00]	**0.003**
Neutrophil count (10^9^ cells/L)		4.16 [3.40, 5.52]	6.66 [4.91, 8.95]	**< 0.001**
Lymphocyte count (10^9^ cells/L)		1.58 [1.23, 1.93]	1.44 [1.17, 1.77]	0.068
Monocytes count (10^9^ cells/L)		0.48 [0.36, 0.65]	0.54 [0.42, 0.74]	**0.009**
HGB (g/L)		140.00 [131.00, 151.00]	139.50 [124.50, 153.25]	0.609
SII		520.33 [372.69, 783.71]	996.82 [595.95, 1565.71]	**< 0.001**
SIRI		1.36 [0.81, 2.00]	2.77 [1.37, 4.60]	**< 0.001**
Urea (mmol/L)		5.40 [4.40, 6.70]	5.20 [4.20, 6.12]	0.157
eGRF (mL/min/1.73 m^2^)		87.52 [69.16, 102.61]	82.39 [61.44, 107.08]	0.876
Echocardiography				
LVEF (%)		57.00 [52.00, 61.00]	55.00 [43.00, 61.00]	**0.022**

*Note:* Bold values indicate statistically significant differences between the two groups.

Abbreviations: BMI, body mass index; DBP, diastolic blood pressure; HDL‐C, high density lipoprotein cholesterol; HGB, hemoglobin; HR, heart rate; hs‐TNT, hypersensitive troponin; LDL‐C, low‐density lipoprotein cholesterol; LVEF, left ventricular ejection fraction; RBC, red blood cell; SBP, systolic blood pressure; SII, systemic inflammatory index; SIRI, systemic inflammatory response index; TC, serum total cholesterol; TG, triglycerides; WBC, white blood cell.

**Figure 3:**
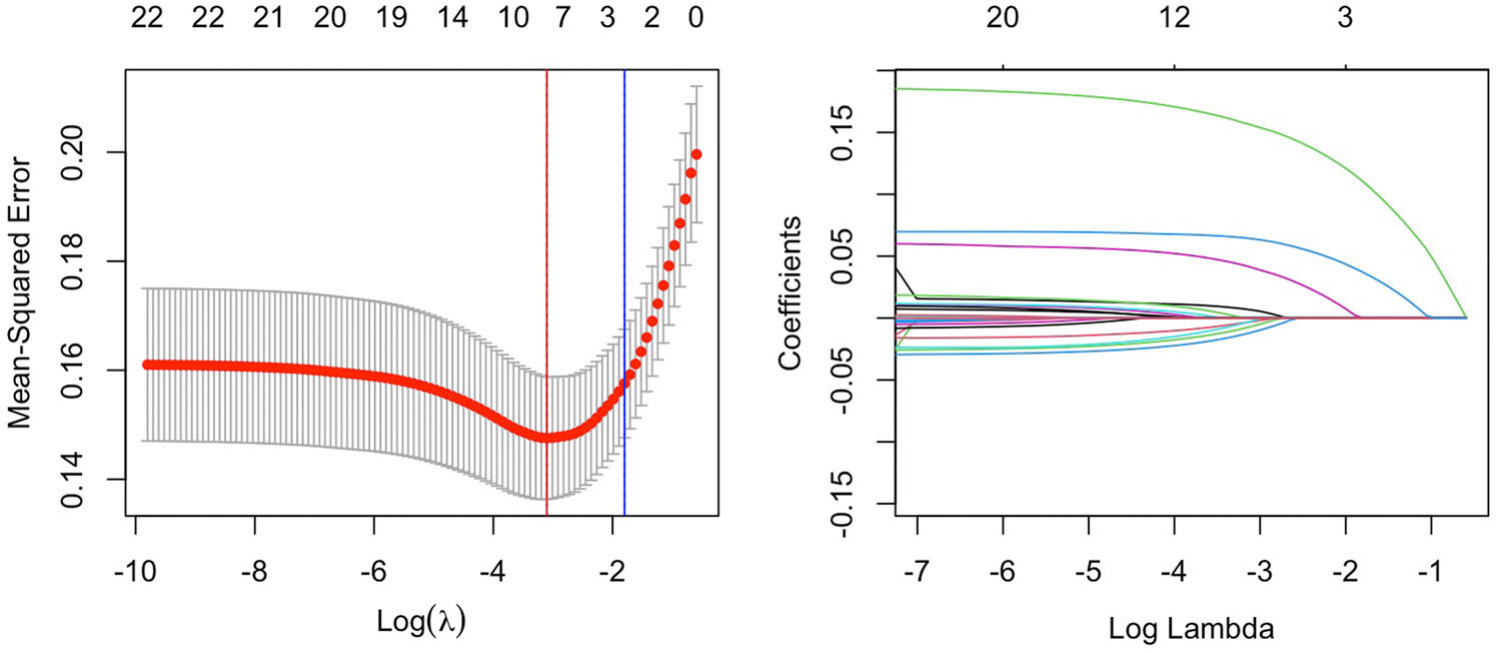
Feature selection based on LASSO regression.

**Figure 4:**
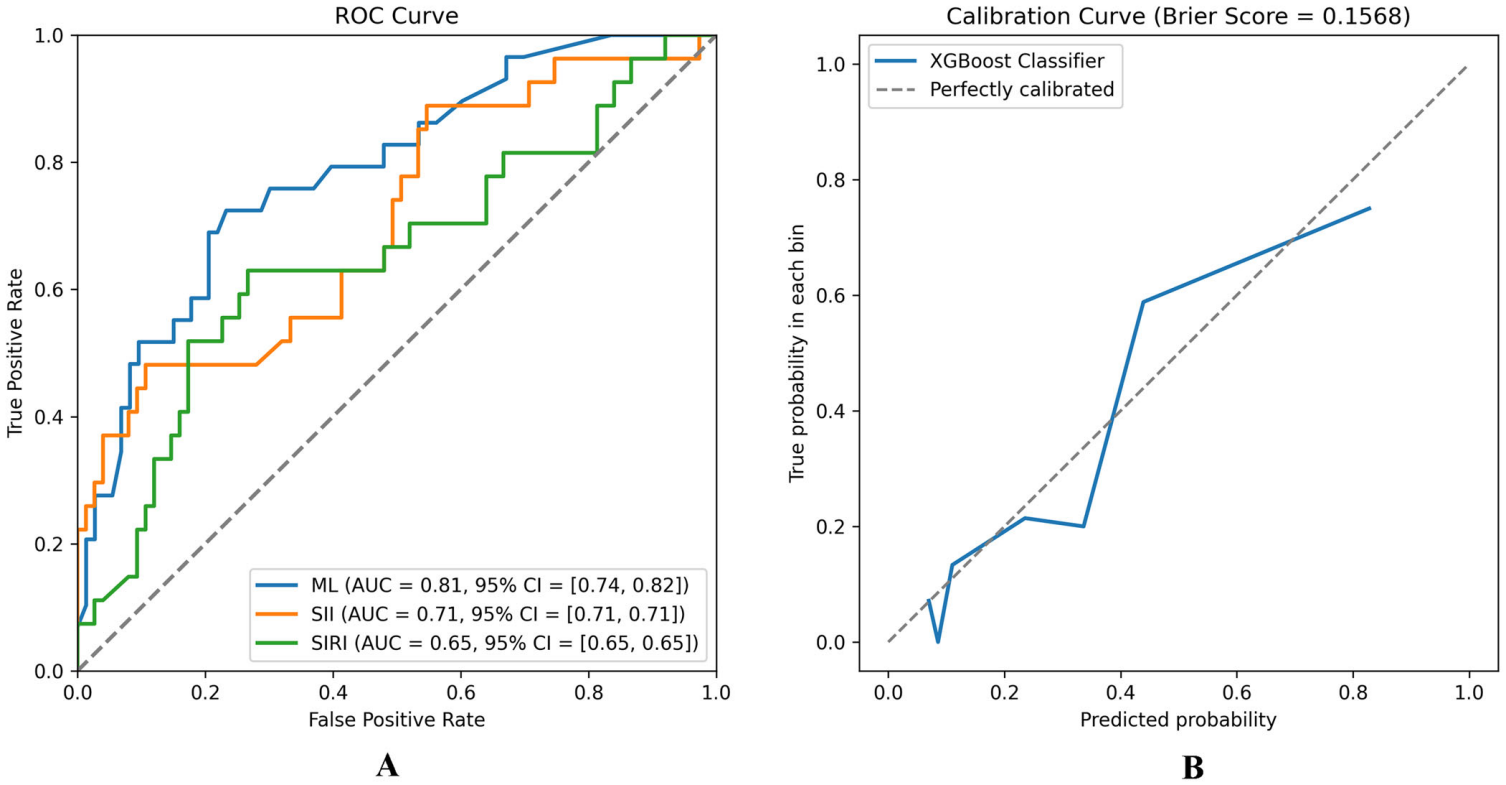
Evaluation of the predictive performance of the ML model. (A) The ROC curves comparing the ML model with using only SII or SIRI; (B) The calibration curve of ML model.

Figure 3 illustrates the results of the LASSO regression Initially, the eight features corresponding to the minimum λ value (BMI, smoking history, presence of hypertension, presence of diabetes, SII, SIRI, serum creatinine levels, and LDL‐C) were utilized to construct the machine learning model. Patients were randomly assigned to training and validation sets at a ratio of 3:1 and grid search was used to find the optimal model parameters, employing fivefold cross‐validation. The performance of the model in predicting NPR and PR was evaluated using ROC curves, and compared with models using only SII or SIRI for prediction of AUC values (Figure 4).

The ML model integrating clinical baseline characteristics, SII, and SIRI demonstrated a higher AUC value (AUC = 0.81, 95% CI = [0.73, 0.82], Figure 4A) compared to models using only SII (AUC = 0.71, 95% CI = [0.53, 0.80]) or SIRI (AUC = 0.65, 95% CI = [0.53, 0.73]). Supporting Material Table 2 provides accuracy, sensitivity, specificity, and F1 score of the model. In the calibration analysis, the machine learning model showed good calibration (Brier Score = 0.1544, Figure 4B).

**Figure 5:**
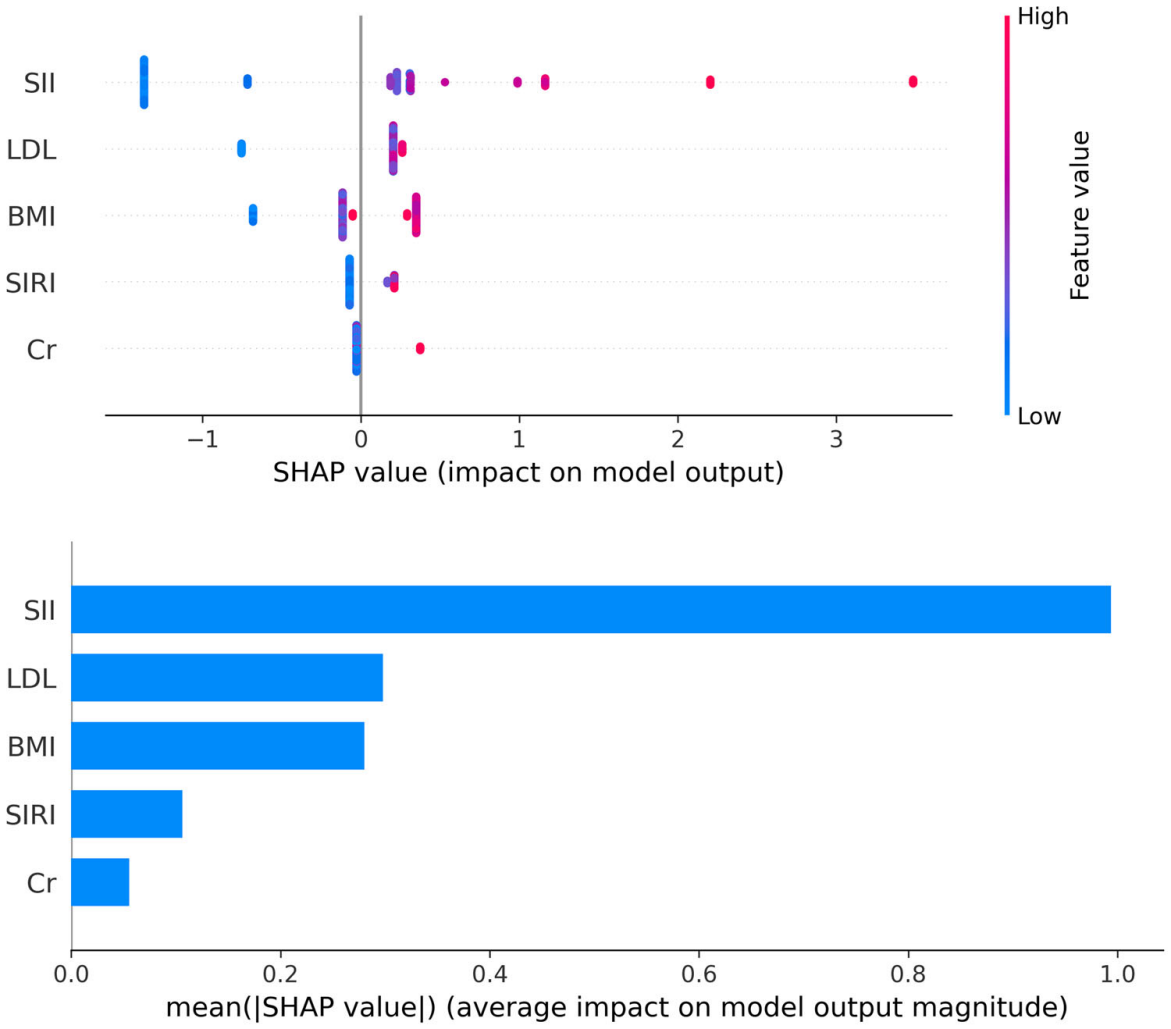
Feature importance of the ML model assessed by SHAP value.

SHAP values revealed feature rankings and the distribution of each feature's impact on the XGBoost model output. The top five features are SII, LDL, SIRI, and serum creatinine levels (Figure 5). Compared to SIRI, SII contributed more significantly to the model output.

## 4 Discussion

PR is a critical event leading to AMI. Current research indicates that PR significantly impacts patient prognosis, with higher rates of major adverse cardiac events (MACE) observed in patients experiencing PR [17]. Advances in coronary intravascular imaging techniques such as OCT have enabled detailed observation of vascular morphology and histological characteristics, including PR [18]. Most existing PR prediction models rely on plaque imaging [19, 20] and may not identify high‐risk individuals early enough.

This study aimed to explore the predictive ability of an ML model integrating clinical features, SII, and SIRI for coronary PR. Early prediction of PR risk is crucial for guiding clinical decisions and reducing adverse events. Our research applied ML methods to integrate clinical features and inflammatory indices to construct a predictive model for identifying patients at risk of PR. Our model primarily focuses on the inflammatory process, as inflammation is a key driving factor for PR. Inflammatory markers such as SII and SIRI are associated with plaque instability, reflecting immune activation within the plaque. Inflammation leads to thinning of the fibrous cap, making the plaque more prone to rupture. By concentrating on measurable inflammation, our model aims to capture the underlying mechanisms of PR.

We included 337 patients presenting with chest pain symptoms who underwent coronary angiography and OCT examination at our hospital. There were no statistically significant differences in baseline characteristics such as age and gender between the two groups. Smoking history, hypertension, and diabetes are known high‐risk factors for CVD. Evidence suggests that smoking can destabilize plaques [21], while diabetes and hypertension predispose coronary plaques to increased vulnerability [22, 23, 24]. The PR group exhibited a higher prevalence of smoking history, diabetes, and hypertension compared to the NPR group. Although no statistical differences were observed, based on the results of LASSO regression, we included these traditional cardiovascular disease risk factors as features in the model.

In laboratory examinations, the PR group exhibited higher levels of LDL‐C (*p* = 0.008, Table 1) and TC (*p* = 0.045, Table 1) compared to the NPR group. Prolonged elevation of LDL‐C levels is a determinant factor in the development and progression of atherosclerotic plaques [25, 26]. Hyperlipidemia contributes to plaque instability, leading to PR, fissures, ulceration, and subsequent intravascular thrombosis, culminating in acute clinical manifestations of AMI [27]. Additionally, patients in the PR group had significantly higher levels of troponin (*p* < 0.001, Table 1), supporting PR as a crucial pathological process leading to acute myocardial infarction [28].

The immune system and inflammatory responses are closely associated with PR. Impaired phagocytic capacity of engulfing cells can lead to accumulation of apoptotic and necrotic tissues within plaques, resulting in enlargement of necrotic cores, narrowing of lumens, exacerbation of plaque inflammation, and ultimately triggering PR [29]. In this study, the PR group exhibited higher levels of traditional inflammatory markers compared to the NPR group, characterized by elevated white blood cell count (*p* < 0.001, Table 1), neutrophil count (*p* < 0.001, Table 1), and monocyte count (*p* = 0.009, Table 1).

Novel inflammatory immune response markers, SII and SIRI, integrate various blood cell types and effectively reflect both local immune responses and systemic inflammation levels [30]. SIRI incorporates three subtypes of blood cells (neutrophils, lymphocytes, and monocytes), reflecting the balance between inflammation and immune response [31]. Studies have also indicated associations between SII, SIRI, and CVD prognosis [32]. In this study, patients in the PR group demonstrated higher SII and SIRI levels (*p* < 0.001, Table 1, Figure 2), consistent with findings by Dziedzic et al [33]. SII and SIRI show significant potential in predicting PR.

However, using only SII or SIRI as singular predictors may lead to insufficient information and overlook factors such as interaction effects, limiting the predictive and generalization capabilities of models [34]. ML has shown great promise in medical applications, particularly in handling large‐scale, high‐dimensional data [35]. ML algorithms are more flexible and capable of handling large‐scale, high‐dimensional data [36]. Due to their ability to handle complex data and features, discover nonlinear relationships, and possess adaptability and generalization capabilities, they have advantages in processing multidimensional data [37].

This study developed a machine learning model comprising eight features, including SII and SIRI, to predict PR. Model interpretability is a critical issue in ML, especially with high‐dimensional datasets where models with numerous features, while potentially accurate, can become complex and challenging to interpret [38]. We improved the model's interpretability and predictive efficacy by employing LASSO regression for feature selection of relevant laboratory tests and baseline data. The comprehensive model based on the XGBoost algorithm exhibited good predictive performance (AUC = 0.81, 95% CI = [0.73, 0.82], Figure 4A). The AUC value for prediction using only SII (AUC = 0.71, 95% CI = [0.53, 0.80]) was lower than that of the ML model. Similarly, using only SIRI for prediction showed lower AUC value (SIRI, AUC = 0.65, 95% CI = [0.53, 0.73]). These results underscore that PR is a multifaceted pathophysiological process [39]. Additionally, XGBoost demonstrated good calibration (Figure 4B).

Based on the XGBoost algorithm, our predictive model further utilized SHAP analysis to assess information gain (Figure 5). The results indicate that the top five features are SII, LDL, SIRI, and serum creatinine levels. Interestingly, SII contributed more significantly to the model compared to SIRI. We believe this may be related to the differences in the calculations of SII and SIRI, with SII incorporating platelet count. The interaction between the coagulation system and the arterial wall is a crucial foundation for the development of peripheral atherosclerotic lesions and thrombosis. Coagulation operates at multiple levels, intersecting and synergizing with inflammatory pathways [40]. Platelets are traditionally known for their role in thrombosis, yet they also have a significant impact on the inflammatory process. Recent studies increasingly recognize the interactions between platelets, immune cells, and endothelial cells as key factors in vascular injury and plaque instability. This phenomenon is referred to as “thromboinflammation,” a term that describes the crosstalk between thrombosis and inflammation [41]. Additionally, the interactions between platelets, monocytes, and neutrophils reflect the extent of endothelial damage [42].

We found that SII, which includes platelet count, contributed more significantly to the model than SIRI, aligning with recent advancements in the understanding of thromboinflammation [40, 41]. The association between platelets and immune responses is critical, as platelet activation and aggregation exacerbate inflammation within atherosclerotic plaques, ultimately leading to plaque rupture. However, whether SII is a superior predictor of myocardial infarction and adverse cardiovascular events compared to SIRI requires confirmation through larger‐scale randomized controlled studies.

This study established a ML model for predicting PR by combining clinical baseline characteristics with SII and SIRI. The predictive performance was compared using ROC curves against individual features. Enhancing model interpretability through LASSO regression feature selection methods, our predictive model holds promise for primary care physicians to identify high‐risk PR patients promptly, enabling more accurate clinical decisions.

Nevertheless, the study has limitations. It is based on retrospective research, introducing potential selection biases, and lacks external validation due to its single‐center design. Additionally, the study focused solely on predicting PR occurrence without assessing prognostic differences between patient groups. The sample size in this study is relatively small, consisting of only a few hundred patients, which may limit the external validity of the findings. Future prospective studies involving larger, multicenter cohorts are needed to validate the generalizability of our model.

In summary, we developed and validated a novel machine learning model that integrates multidimensional features, including SII and SIRI, to predict the occurrence of PR. The ML model demonstrates a high AUC value and shows advantages over single‐feature prediction. Our study highlights the potential of ML in predicting PR occurrence, offering promise in assisting physicians to identify high‐risk patients promptly and intervene to improve patient outcomes.

## Author Contributions

Xi Wang and Qianhang Xia performed the research. Xi Wang, Qianhang Xia, Shuangya Yang, and Chancui Deng designed the research study. Xi Wang, Qianhang Xia, Shuangya Yang, Chancui Deng, Ning Gu and Youcheng Shen collected the relevant data. Xi Wang, Qianhang Xia analysed the data. Xi Wang and Qianhang Xia wrote the paper. Zhenglong Wang, Bei Shi and Ranzun Zhao supervised the research. All authors have read and approved the final manuscript.

## Supporting information

Supporting information.

## Data Availability

The data supporting the findings of this study are available from the corresponding author upon reasonable request.
