# Supplementary material for: Machine Learning‐Based Immuno‐Inflammatory Index Integrating Clinical Characteristics for Predicting Coronary Artery Plaque Rupture
Source: Immun Inflamm Dis. 2025 Apr 7;13(4):e70162. doi: 10.1002/iid3.70162 (PMC11973732; doi:10.1002/iid3.70162)
Supplement: Supplementary file 1 — Supporting information. [file IID3-13-e70162-s001.docx]

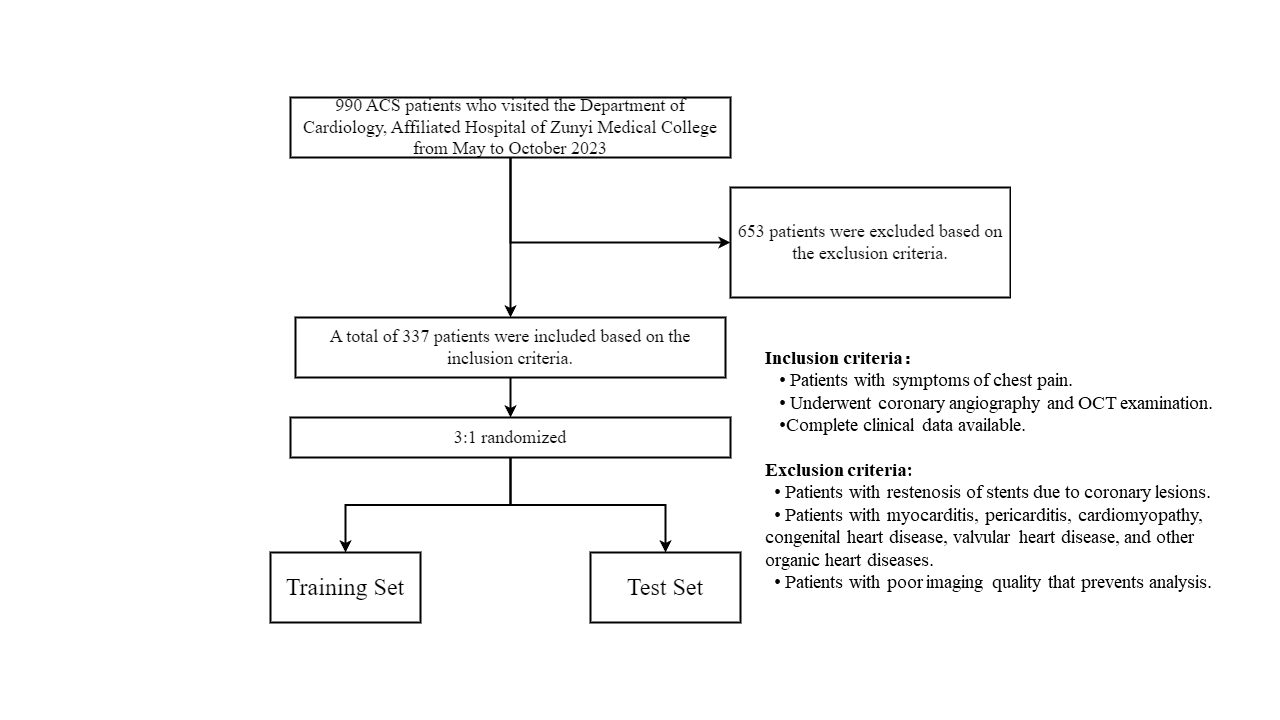


Supplementary Figure 1: Inclusion and exclusion criteria for patient

Supplementary Table 1: Detailed parameters of the Xgboost algorithm:

| Model | parameters |
| --- | --- |
| XGBoost | n_estimators=30, scale_pos_weight=2.06, gamma=1, max_depth=2 |

Supplementary Table 2: Performance evaluation of models:

| Model | AUC（95%Cl） | SEN（95%Cl） | SPE（95%Cl） | ACC（95%Cl） | PPV（95%Cl） | NPV（95%Cl） | F1 score（95%Cl） |
| --- | --- | --- | --- | --- | --- | --- | --- |
| ML | 0.81(0.73,0.82) | 0.54 (0.42,0.61) | 0.94 (0.88,0.97) | 0.79(0.68,0.84) | 0.71(0.62,0.79) | 0.88(0.69,0.85) | 0.57(0.47,0.66) |
| SII | 0.71(0.61,0.81) | 0.30(0.22,0.40) | 0.97(0.92,0.99) | 0.78(0.70,0.86) | 0.80(0.71,0.87) | 0.79(0.70,0.86) | 0.43(0.34,0.53) |
| SIRI | 0.75(0.65,0.85) | 0.19(0.09,0.23) | 0.92(0.85,0.96) | 0.72(0.62,0.79) | 0.40(0.31,0.50) | 0.75(0.65,0.82) | 0.22(0.15,0.31) |

AUC: Area Under the Curve; SEN: Sensitivity; SPE: Specificity; ACC: Accuracy; PPV: Positive Predictive Value; NPV: Negative Predictive Value.
